# Treatment of lupus erythematosus with fumaric acid ester derivatives: two case-reports

**DOI:** 10.1186/1479-5876-9-S2-P15

**Published:** 2011-11-23

**Authors:** Deepak MW Balak, H Bing Thio

**Affiliations:** 1 Dept. of Dermatology, Erasmus Medical Center, Rotterdam, The Netherlands

## Introduction

Fumaric acid ester derivatives, or fumarates, have immunomodulating properties [1]. For over four decades fumarates have been applied in the treatment of psoriasis with proven efficacy and long-term safety [2,3]. More recently, fumarates have been assessed in multiple sclerosis [4]. Given their favorable safety-profile, fumarates could be of value in the treatment of other immune-mediated inflammatory diseases.

## Aim

To assess the response of fumarates in lupus erythematosus.

## Methods

Two case-reports of patients with lupus erythematosus who were treated with fumarates. Treatment was carried out with enteric-coated tablets containing 120 mg dimethylfumarate and 95 mg monoethylfumarate-salts. The dosage was gradually increased up to 6 tablets daily. Patients were followed up regularly with clinical evaluations, laboratory tests, and urine analysis.

## Results

A 35-year old female patient presented with an erythematous indurated swelling of the nose that was present for 1 year. Histology of a skin biopsy was consistent with the diagnosis lupus timidus. Treatments included hydroxychloroquine 200 mg two times a day for four months and intralesional corticosteroid injections, but both treatments were without effect. Therefore, fumarates treatment was started. After 3 months the skin lesions resolved completely. At one year follow-up the clinical situation was stable with 4 tablets daily. Adverse events were nausea, diarrhea, and mild leukopenia and proteinuria, but these resolved gradually.

A 24-year old female patient presented with erythematous scaly plaques, Raynaud's phenomenon, alopecia, and oral ulcera with positive antinuclear antibodies. She was diagnosed with systemic lupus erythematosus. Treatments with prednison, hydroxychloroquine, and cyclosporin were ineffective. Therefore, fumarates in combination with prednisone 10 mg was started, which resulted in improvement of the skin lesions. Because of a pregnancy wish, fumarates was stopped. Re-treatment with fumarates had an equally good effect within 3 months. Adverse events included tiredness, pruritus, abdominal cramps, and transient proteinuria.

## Conclusions

Fumarates seem beneficial in the treatment of lupus erythematosus not responding to conventional systemic treatments. The short-term side effect profile is similar to that in psoriasis patients.
